# KAT tales: Functions of Gcn5 and PCAF lysine acetyltransferases in SAGA and ATAC

**DOI:** 10.1016/j.jbc.2024.107744

**Published:** 2024-09-01

**Authors:** Sharon Y.R. Dent

**Affiliations:** Department of Epigenetics and Molecular Carcinogenesis, University of Texas M.D. Anderson Cancer, Center for Cancer Epigenetics, University of Texas M.D. Anderson/UTHealth Houston Graduate School of Biomedical Sciences, Houston, Texas, USA

**Keywords:** histone acetylation, Gcn5, PCAF, SAGA, ATAC, cancer, neural degeneration

## Abstract

The Allis group identified Gcn5 as the first transcription-related lysine acetyltransferase in 1996, providing a molecular “missing link” between chromatin organization and gene regulation. This review will focus on functions subsequently identified for Gcn5 and the closely related PCAF protein, in the context of two major complexes, SAGA and ATAC, and how the study of these enzymes informs long standing questions regarding the importance of lysine acetylation.

A role for histones in regulating RNA synthesis was first reported in 1963 by Allfrey *et al.* (1). Two years later, these authors presented evidence that posttranslational modifications, including both histone acetylation and methylation, had strong regulatory potential for RNA transcription (2). Almost immediately the hunt for enzymes that regulate these modifications began, with a report by Nohara *et al.* in 1966 that biochemical fractionation of pigeon liver extracts yielded an activity that could acetylate histones *in vitro* (3). Almost three decades would pass before specific proteins were identified with histone acetyltransferase (HAT) activity. In the meantime, scores of studies demonstrated that increased histone acetylation correlated with increased chromatin accessibility and gene activity, and conversely, that decreased acetylation correlated with ‘closed’ chromatin states and gene repression (4). Establishing mechanisms underlying these correlations, though, required identification of enzymes responsible for acetylation and deacetylation.

In 1995, the Sternglanz group identified Hat1 as an H4 specific HAT using an elegant combination of yeast genetics and biochemistry (5). Hat1 and associated proteins are important for histone deposition onto newly replicated DNA during S phase (6). The first transcription-related HAT was identified in *tetrahymena* the following year by the Allis group (7). The *tetrahymena* HAT, p55, was homologous to the yeast protein Gcn5, which had previously been identified as a transcriptional coactivator (see Ref. (8) for review), providing the first direct molecular link between histone acetylation and gene activation. Serendipitously, a human homolog of Rpd3, a yeast repressor protein, was also reported as the first histone deacetylase in 1996 by the Schreiber group (9), further cementing changes in histone acetylation states with changes in gene expression.

Why did it take so long to identify these enzymes? One issue had to do with the difficulty in measuring acetyltransferase activity *in vitro*. Assays relied on incorporation of labeled acetyl moieties from ^3^H or ^14^C labeled acetyl CoA into histones (10). Although overall acetylation activity could be monitored by scintillation counting, demonstrating acetylation of a particular histone often took long exposures of autoradiograms of histones separated by electrophoresis. Even when investigators succeeded in identifying a biochemical fraction with HAT activity, they were not able to assign that activity to a specific protein in the fraction. Brownell *et al.* were able to overcome these problems by using macronuclei with a highly amplified genome isolated from *tetrahymena*, which provided biochemical fractions rich with HAT activity, and an “in gel” assay (11) adapted from studies of kinases that allowed identification of which exact band in the fraction housed HAT activity upon exposure to labeled acetyl-CoA. Further biochemical isolations, together with genetic studies, revealed that Gcn5 is part of a highly conserved, multisubunit complex, SAGA, which confers substrate specificity for Gcn5 as well as full activity (12).

The identification of Gcn5 (and other Gcn5-related N-acetyltransferases (GNATs)) and Hat1 opened the door for sequence- and activity-based identification of additional lysine acetyltransferase (KAT) proteins, including the MYST family (typified by Moz, Sas2, and Tip60), Rtt109, and p300/CBP (reviewed in (13, 14, 15, 16)). The sequence of the KAT domain differs in these five KAT families, and they associate with different protein partners, which recruit these activities to specific genomic loci and/or contribute to substrate specificity (13). These enzymes were renamed as lysine (K) acetyltransferases (KATs) to reflect their widespread activity on nonhistone proteins in addition to histones (17). KATs have proven important both for normal cellular and developmental processes as well as disease states, as discussed in several excellent previous review articles (18, 19, 20, 21). This review will focus on the functions of two major evolutionarily conserved Gcn5-containing complexes, SAGA and ATAC.

## SAGA and ATAC

**Figure 1:**
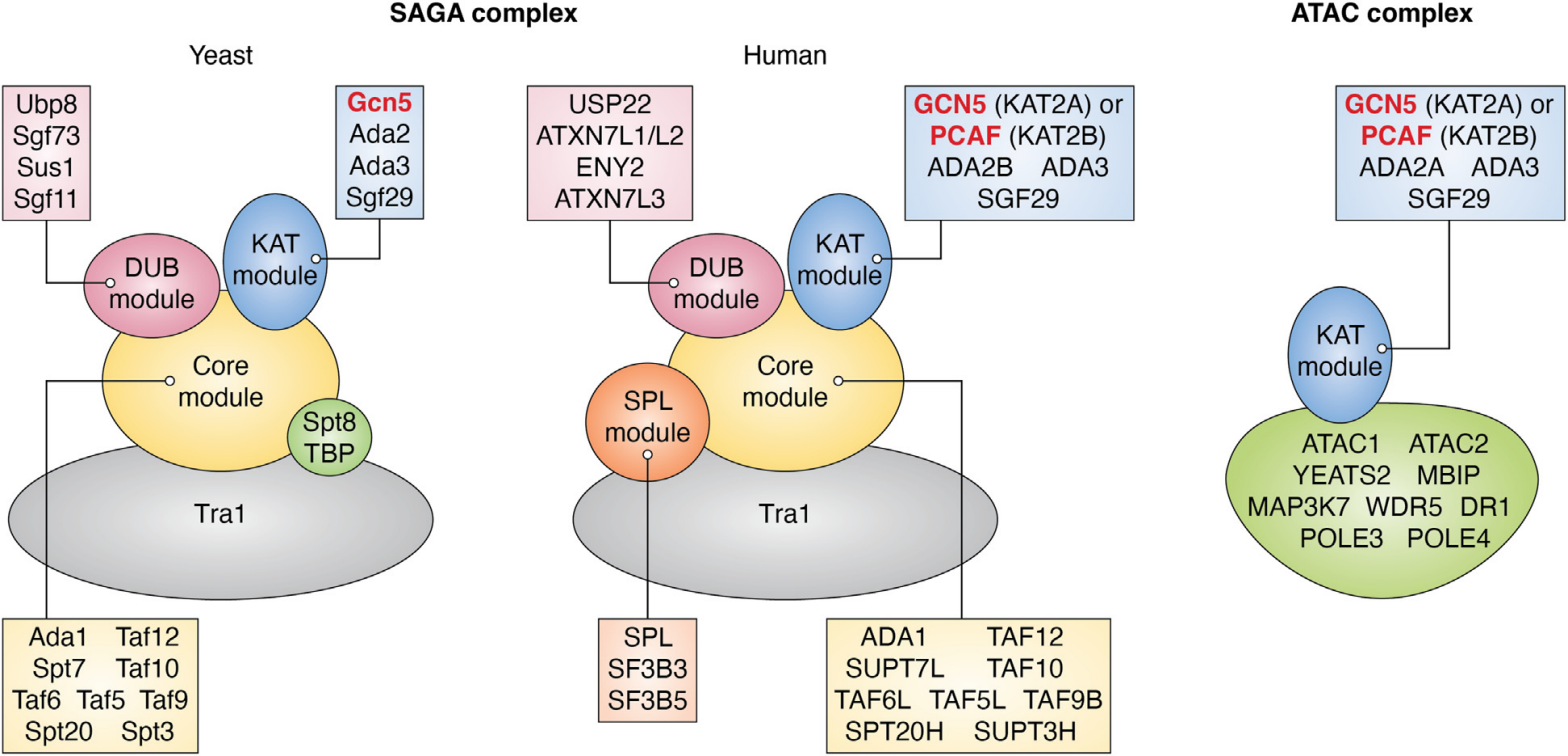
**Gcn5 and PCAF provide KAT activity to SAGA and ATAC.** Schematic representations of SAGA and ATAC, two major complexes that include Gcn5 or PCAF in a mutually exclusive way. Yeast and human versions of SAGA are shown, to highlight both evolutionary conservation of modules and subunits, as well as differences, as discussed in the text. ATAC, ADA two A containing; KAT, lysine (K) acetyltransferase; PCAF, P300/CBP-associated factor.

Gcn5-related N-acetyltransferases share a unique structure, comprising a superfamily that stretches across plants, animals, and microbes (12, 13, 22, 23, 24). Gcn5 (also known as KAT2A (17)) has limited activity on its own, *in vitro*. *In vivo*, Gcn5 associates with transcriptional adaptor proteins Ada2, Ada3, and Sgf29 to form an active KAT module (12, 25, 26). Sgf29 docks the KAT module into the larger SAGA complex, which also includes a deubiquitylase (DUB) module, a module containing the large Tra1/TRRAP protein that interacts with sequence-specific DNA binding proteins, and a core module that bridges interactions between SAGA components (26, 27) (Fig. 1).

Ada2 and Ada3 were recognized to be important for Gcn5 functions in the regulation of transcription even before Gcn5 was identified as a KAT (28, 29, 30). These proteins were named for their adaptor functions in bridging interactions between transcriptional activators and the RNA pol II transcription machinery. Both are also important for activity of the KAT module toward nucleosomes, as well as other proteins (12, 23, 25, 31). In addition to SAGA, The ADA proteins constitute a small complex together Gcn5 and Sgf29 in both yeast and mammalian cells (32). Two Ada2 paralogs exist in metazoans, ADA2A and ADA2B, which nucleate the ADA two A containing (ATAC) and SAGA complexes, respectively (26, 33) (Fig. 1). Outside of the KAT module, ATAC is comprised of a completely different set of proteins than SAGA, and includes a second KAT enzyme, ATAC2, as well as YEATS2, ZZZ3, MBIP, WDR5, and NC2beta (33) (Fig. 1). Previous reviews provide additional details on organization and structure of these complexes (26, 34).

Loss-of-function experiments in mES cells reveal differential effects of SAGA and ATAC depletion on gene expression and histone acetylation patterns (35), consistent with earlier studies in human cells that indicated these complexes regulate different sets of genes, through different mechanisms (36). Complete loss of genes encoding Ada2a or Ada2b causes lethality in flies, but at different developmental stages, again with differing effects on histone acetylation (37, 38), further establishing the distinct functions of SAGA and ATAC.

Mammals also contain a paralog of GCN5, P300/CBP-associated factor (PCAF) (KAT2B), which associates with the SAGA and ATAC complexes mutually exclusively of GCN5 (39). Interestingly, mammalian GCN5 has a long amino terminal domain not found in yeast that is homologous to PCAF and that is important for acetylation of nucleosomes (40). Biochemically, GCN5 and PCAF appear to be very similar, but mutations of the genes encoding these KATs yield quite different phenotypes in mice. Loss of GCN5 leads to embryonic lethality, whereas PCAF null mice survive to adulthood with no overtly abnormal phenotypes (41, 42). Deletion of both GCN5 and PCAF, however, leads to even earlier lethality in mice, indicating the two KATs share some functions in early development. Whether and under what conditions GCN5 and PCAF do or do not share functions in disease states could prove relevant to future development of therapies for human diseases.

The embryonic lethal phenotypes of GCN5 mutations likely reflect loss of function of both SAGA and ATAC. Deletion of the ATAC2 KAT also results in early embryonic lethality in mice, but with differing impacts on lineage specification and development than Gcn5 deletion (43). Further definition of the division of labor between SAGA and ATAC will likely be important in both developmental and disease settings.

## SAGA: More than a KAT

Definition of yeast Gcn5 as a KAT together with its partnership with the Ada adaptor proteins, immediately led to models connecting Gcn5/Ada recruitment to promoter regions to acetylate histones and open chromatin, followed by stabilization of interactions between activators, RNA polymerase, and general transcription factors by Ada2/Ada3 (8). As more refined views of SAGA structure and composition became available, it became clear that the different modules of the SAGA complex coordinate multiple steps in the process of gene transcription, including not just acetylation, but also deubiquitylation of H2B, and in yeast, deposition of the general transcription factor, TATA binding protein (TBP) (44, 45, 46). SAGA has also been linked to buffering transcriptional noise (47).

Structural studies revealed differences in SAGA organization in yeast and human cells that refine its functions in these organisms (27, 48, 49) (Fig. 1). Mammalian SAGA lacks Spt8, which is critical in yeast for TATA binding protein interaction (50). Human and fly SAGA contain a module not present in yeast that includes two proteins, that participate in RNA splicing in the context of a submodule of the U2 small ribonucleoprotein (51, 52). The functions of these splicing factors in the context of SAGA are not clear, but it is tempting to think that they might coordinate RNA transcription with RNA processing. Interestingly the placement of the KAT and DUB modules of both the yeast and the human complexes could not be resolved in the structures, indicating flexible or dynamic associations of these enzymes with SAGA. Their general location could be inferred from the location of tethering subunits (*e.g.*, Sgf73/ATXN7 for the DUB module and TAF6L/SUPT7L or Ada3 for the KAT module). Previously defined structures of the catalytic modules provide clear pictures for how their subunits interact (53, 54, 55). The flexibility of the interactions of the KAT and DUB modules with the greater SAGA complex has been proposed to facilitate interactions with multiple histones once recruited to a given locus.

## Importance of Gcn5 KAT activity *in vivo*

**Figure 2:**
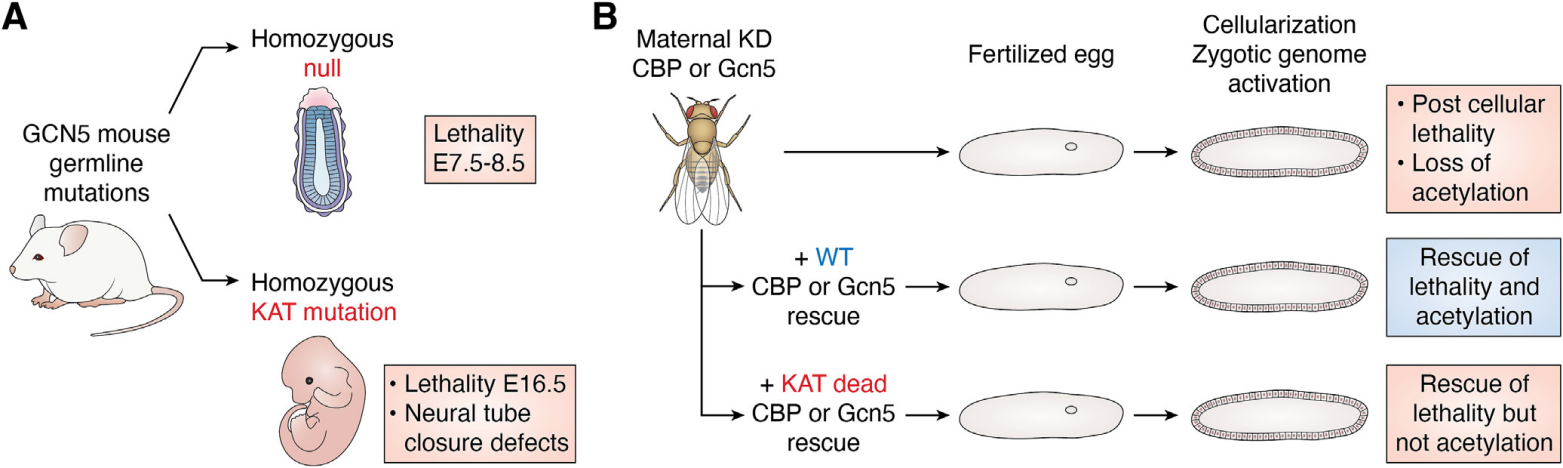
**KAT dependent and independent functions *in vivo*.***A*, differences in phenotypes observed in *GCN5* null mice and mice bearing mutations in the GCN5 catalytic center reveal that GCN5 has some KAT independent functions early in development, but that KAT activity is required for neural tube closure and embryo survival. *B*, postcellular lethality of fly embryos upon maternal depletion of either GCN5 or CBP can be fully rescued by KAT mutant forms of the enzymes, even in the absence of restoration of H3 acetylation patterns during zygotic gene activation. These results indicate that both KATs have essential KAT-independent functions during ZGA. KAT, lysine (K) acetyltransferase; ZGA, zygotic gene activation.

Since SAGA impacts multiple steps in gene transcription, one might wonder how important the KAT activity of Gcn5 is relative to these other functions (44). Genetic studies provide some clues. Mutation of residues in Gcn5 critical for KAT activity in yeast yields a phenotype that mimics that of *gcn5* null cells (53), suggesting KAT activity is essential for Gcn5 functions. Mice bearing point mutations in analogous residues of the mammalian GCN5 catalytic center develop until midgestation (56), in contrast to the earlier lethality, just after gastrulation, caused by complete GCN5 loss (41, 42) (Fig. 2). Importantly, the KAT mutations did not impact GCN5 protein levels or the ability of GCN5 to incorporate into SAGA in mice. These results indicate that both GCN5 KAT activity is important for normal mouse development, especially in the process of neural tube closure midgestation, and that GCN5-containing complexes have additional KAT-independent functions in early development. Later, studies revealed that complete loss of GCN5 destabilizes association of the DUB module with SAGA, in both yeast (57) and mice (58). At least part of the more severe phenotype of the *GCN5* null mutation was due to decreased activity of the dissociated DUB module toward a nonhistone substrate required for telomere capping and maintenance of genome integrity, ultimately causing large scale apoptosis and cell death (58). These findings highlight the physical and functional interdependence of SAGA modules *in vivo*. Loss of GCN5 KAT activity did not have this effect on DUB activity. The severe neural tube closure phenotype caused by the KAT mutations was later determined to be related to changes in acetylation of a nonhistone protein important in retinoic acid signaling, rather than to changes in histone acetylation (59). GCN5 KAT activity is also critical for maturation of craniofacial chondrocytes in mice, again independently of changes in H3K9ac. Rather GCN5 activity is required for proper mammalian target of rapamycin complex 1 signaling (60). Altogether, these studies highlight the importance of the KAT activity of GCN5, but also emphasize its role in multiple processes beyond histone acetylation and transcriptional regulation.

Interestingly, a recent study revealed that Gcn5 and CBP (also known as nejire in flies), but not their KAT activities, are required for zygotic gene activation (ZGA) in flies (61) (Fig. 2). Loss of each KAT impacted distinct gene expression progression programs. CBP was required for proper expression of genes involved in germ layer commitment and gastrulation, including important developmental genes such as *twist, hh, ci,* and *wg*. Gcn5, in contrast, was required for expression of housekeeping genes and of genes involved in postembryonic developmental processes. Also, Gcn5 was found to be crucial for acetylation of H3K9 at ZGA, and CBP was required for acetylation of H3K18 and H3K27. Strikingly, even though these modifications were present at all actively transcribed genes during ZGA, loss of these marks upon mutation of the KAT catalytic centers of Gcn5 or CBP, respectively, was not sufficient to alter ZGA. Moreover, expression of the KAT mutants rescued the depletion phenotypes, raising questions as to the importance of these specific acetylation events in gene activation in this setting (62). CBP provides a scaffold for interactions with multitudes of proteins (16), so perhaps this coordination function, rather than KAT activity, is especially important during ZGA. Gcn5, as part of SAGA and ATAC, contributes to many steps in gene activation beyond acetylation, as described above, which may outweigh the importance of H3K9ac acetylation in ZGA.

These findings highlight a central, long-standing issue in the study of histone acetylation: how important is acetylation at specific lysines *versus* overall histone acetylation levels to the regulation of gene expression? Before the availability of site-specific antibodies, changes in overall acetylation levels under different cellular or physiological conditions was assessed in isolated histones by gel electrophoresis (see Refs. (10, 63) for examples). Such studies connected “hyperacetylated” states with increased chromatin accessibility and gene activity, indicating concomitant changes in the acetylation states of multiple histones. These classic studies are consistent with more recent studies that link increased acetylation of multiple nearby lysines in histones with not only altered electrostatic interactions with DNA but also with increased affinity of bromodomain reader proteins (64, 65) and accessibility of lysine methyltransferases and methyl-lysine readers (66). The advent of histone acetylation site-specific antibodies in the late 1980s allowed visualization of changing histone states in cells by immunofluorescence, providing the first clues as to when and where specific acetylation events were altered (67, 68). Antibody approaches have become ever more important to defining epigenetic landscapes through chromatin immunoprecipitation genome sequencing and related mapping techniques, as illustrated in the above study focused on ZGA in flies. However, these approaches have limitations that include antibody specificity, limited linear dynamic ranges, and limited ability to detect combinatorial changes in acetylation (69, 70).

Mass spectrometry offers an alternative to defining changes in histone acetylation patterns, as demonstrated by Feller *et al.* (71), who developed elegant approaches to define and to quantify histone acetylation and methylation motifs in fly KC cells. The authors quantified 45 motifs that included four acetylation sites and one methylation site in H4 and seven acetylation and five methylation sites in H3. They then determined how loss of 23 individual KATS or 10 individual KDACs impacted these patterns. Surprisingly, their findings revealed that loss of individual KATs had remarkably little impact on overall acetylation levels in KC cells, although specific acetylation motifs were impacted by loss of particular KATs. Depletion of Gcn5, for example, resulted in decreased levels of H3K9ac in multiple combinatorial motifs (*e.g.*, H3K9Ac and H3K9/K14Ac), but also decreased levels of peptide motifs bearing H3K27Ac and H3K36Ac. Loss of CBP resulted in loss of multiple combinatorial motifs that included H3K27Ac as expected, but also caused decreased acetylation of motifs bearing H3K18 and K36 as well as acetylation of H4K5 and K8. These findings are a good reminder that although particular KATs have become synonymous with specific lysine acetylation events, *e.g.*, CBP/p300 and H3K27Ac, these enzymes actually acetylate a broad range of sites in histones and other proteins. Moreover, Feller *et al.* found that loss of acetylation at particular sites upon loss of an individual KAT was accompanied by increased acetylation and/or methylation at other lysines, in unrelated acetylation motifs, sometimes even on different histones. Decreased abundance in H3K27Ac motifs upon CBP depletion was accompanied by increased abundance of peptides bearing acetylation of H4K12, diacetylation at both H4K12 and K16, or both H3K9 dimethylation and K14acetylation. Loss of H3K23Ac, which was highly abundant in fly KC cells, occurring on 47% of H3, upon depletion of KAT6 was accompanied by increased acetylation of H3K18 as well as several motifs bearing H4K16ac. This unanticipated cross talk suggests that a balance in overall acetylation levels may be critical to gene activation. Notably, no major changes in cell cycle progression or cell growth were observed upon depletion of any of the KATs in the study, so the histone modification changes observed were not likely due to indirect effects on cell division or survival. Unfortunately, mass spec-based approaches cannot map where in the genome changes in acetylation or methylation occur, so it is difficult to know whether such cross talk occurs on a local level, at specific genes. Still, it would be interesting to perform such studies in additional settings including processes such as ZGA to determine if loss of specific acetylation events in the absence of Gcn5 or CBP is accompanied by increased acetylation or methylation of other lysines in H3 or other histones.

## Acylation

Gcn5 activity, like that of other KATs, is dependent on acetyl-CoA, a central metabolite that bridges anabolism, catabolism, and energy production (72). KATs also use other acyl-CoA cosubstrates, and histones are subject to a variety of acylation events (73). The structure of the Gcn5 active site can accommodate smaller acyl-CoA cofactors, such as propionyl-CoA, but not larger acyl moieties (13, 74). Butyryl-CoA actually serves as a competitor inhibitor of Gcn5 activity. A report of Gcn5 involvement in histone succinylation (75) is confounded by these structural restraints, as well as by the propensity for nonenzymatic succinylation of histones in presence of high local levels of succinyl Co-A (13, 76). GCN5/KAT2A was also identified in a knock down screen of 22 KATs to be selectively required for maintenance of malonyl-K levels in K562 cells (77). *In vitro* assays using histone peptides and the isolated catalytic domain of GCN5 revealed malonylation activity toward an H3 tail peptide, but not other core histone tails. *In vivo*, however, H2BK5 was identified as the most prevalent malonylated histone in both mouse liver and brain, making it hard to connect the *in vitro* activity of the GCN5 catalytic domain toward histone peptides directly to changes in malonyl-K *in vivo*. Still, defining the occurrence, levels, and functions of specific acylation events by GCN5 and other KATs is an important, ongoing area of research.

## Links to human disease states

Both SAGA and ATAC have been linked to multiple human conditions. While not all can be covered here, a few examples include cancer, immune responses, neural conditions, and lipodystrophy.

## Gcn5 and cancer

**Figure 3:**
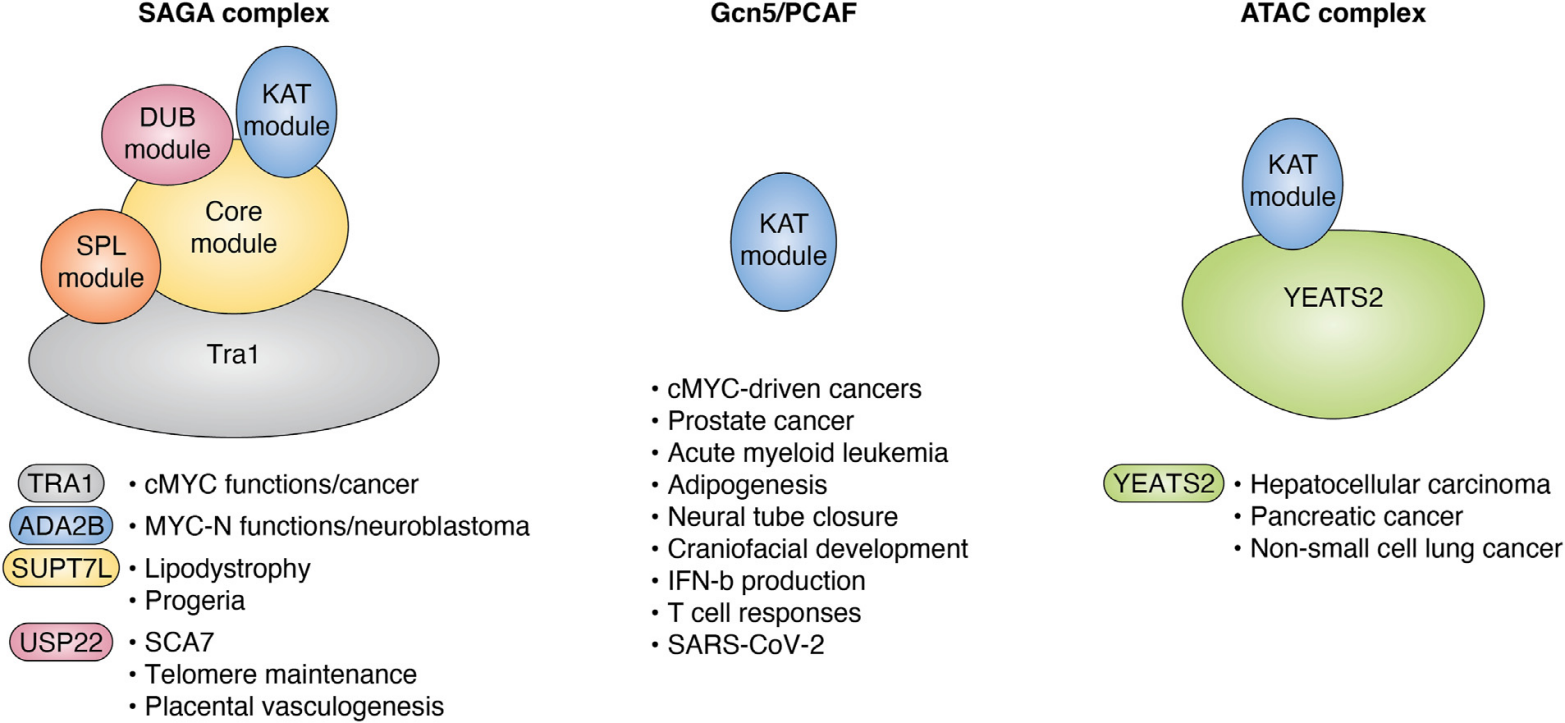
**Human conditions associated with SAGA and ATAC.** GCN5 and PCAF functions have been linked to many processes involved in disease states (*middle*). In many cases, it is not clear whether these functions are executed in the context of SAGA or of ATAC, but mutations in complex-specific components indicate both SAGA (*left*) and ATAC (*right*) are involved in multiple human conditions. ATAC, ADA two A containing; PCAF, P300/CBP-associated factor.

GCN5/KAT2A is implicated in several types of cancer (20, 21) (Fig. 3). SAGA, for example, interacts physically with c-MYC through the TRAPP subunit (78) and the c-MYC protein is stabilized through acetylation by GCN5 or PCAF (79). c-MYC also interacts with other KATs, including p300/CBP and TIP60, and it is not clear how these different activities are coordinated at MYC gene targets (80). The importance of GCN5-MYC interactions is highlighted by the finding that deletion of GCN5 in the Eμ−MYC mouse model of B-cell lymphoma significantly extends mouse survival in concert with destabilization of MYC protein and downregulation of MYC target genes that normally promote proliferation of B cell precursors over differentiation (81).

GCN5 activity and functions have also been linked to other cancers, often mirroring GCN5 functions in normal development processes (20, 82). GCN5/KAT2A loss increases transcriptional “noise” in both stem cells and in acute myeloid leukemia (AML), thereby impacting cell fates (47). KAT2A was also identified as vulnerability in human AML cells in a CRISPR dropout screen (83). Interestingly, though, changes in the levels or distributions of H3K9 acetylation are not always associated with changes in GCN5 KAT module activity, again indicating that other acetylation sites and other substrates may be more important in specific biological settings. For example, GCN5/KAT2A-mediated acetylation of H2AK130 is important in SREBF1 driven cholesterologenic transcription programs that facilitate androgen production in prostate cancer cells (84).

Gcn5 inhibition or depletion impacts both SAGA and ATAC, and so cannot distinguish functions of these complexes in specific cancers. A recent study more directly tied SAGA functions to MYC-N driven neuroblastoma by showing that ADA2B is specifically required for MYC-N driven oncogenic transcription programs (85). Similarly, YEATS2 within the ATAC complex has been linked to non—small cell lung cancers that bear YEATS2 gene amplifications (86). In this setting, the function of YEATS2 as an H3K27ac reader coordinates ATAC-mediated acetylation of H3K9ac at target gene promoters, and disruptions of these functions inhibits tumorigenesis. YEATS2 functions have also been linked to hepatocellular carcinoma (87, 88) and hypoxia responses in pancreatic cancer cells (89). These studies suggest that inhibiting selective inhibition of SAGA and ATAC activities could provide therapeutic benefits.

## Links to immunity

GCN5 functions have been linked to both innate and adaptive immune responses. Both PCAF and GCN5 negatively regulate production of interferon-beta in a KAT independent mechanism involving the TBK kinase (90), and they impact dsRNA accumulation and interferon signaling in intestinal stem cells (91). An interesting connection has also emerged between GCN5 and a SARS-CoV-2 viral protein, encoded by *ORF8*. ORF8 contains peptide sequence, ARKS, which mimics the sequence of histone H3 at both K9 and K27 (92). ORF8 interacts physically with GCN5 and is acetylated by it at this motif. ORF8 also triggers lysosomal destruction of GCN5 and other proteins. Thus, ORF8 may provide a decoy that pulls GCN5 away from its chromatin targets, triggering its destruction and disruption of its functions in transcription and other processes. A stretch of 51 amino acids in ORF8 aligns with a sequence in GCN5/KAT2A (93). How or if this peptide sequence impacts ORF8 or GCN5 functions is not yet clear. Nevertheless, these intriguing observations suggest that diminished GCN5 activity may enhance SARS-CoV-2 infection, perhaps through effects on beta-interferon expression and innate immunity.

GCN5 is important for T-cell responses too. Deletion of GCN5 in T-lymphocytes in mice impairs T-cell activation (94). GCN5 is also required for maturation of CD1-restricted invariant natural killer T cells through acetylation of EGR2, a transcription factor that drives expression of multiple genes needed for invariant natural killer T cells (95). Hopefully future studies will further determine whether alterations of GCN5 functions impact the efficacy of immunotherapies for cancer or inflammatory responses in autoimmune diseases. GCN5/KAT2A has been linked to inflammasome activation in macrophages, which may impact rheumatoid arthritis and inflammatory diseases (96).

## Links to lipodystrophy

Recent studies have identified *SUPT7L* loss-of-function mutations in a patient exhibiting lipodystrophy and features associated with progeria, a condition of premature aging (97). So far, only one individual has been identified bearing *SUPT7L* mutations, but the 1ed gene. SUPT7L is the mammalian homolog of yeast Spt7, and this subunit is integral to SAGA integrity in both organisms (50, 51), as it is a member of the histone-like fold region in the core module (27, 48, 49). The patient’s missense mutations cause a complete loss of SUPT7L, impacting both GCN5- and PCAF-containing SAGA complexes. RNA analyses connect SUPT7L loss with changes in MYC target gene expression, as well as genes involved in DNA repair pathways, fitting with prior connections of these processes with SAGA (51). Increased DNA damage was observed in patient-derived fibroblasts as well as in HeLa cells expressing CRISPR-generated mutations in *SUPT7L*, consistent with loss of genome integrity that is commonly observed in progeroid disorders. Although this study did not provide direct insights into how SAGA loss-of-function might contribute to lipodystrophy, GCN5 and PCAF are required for adipogenesis in mice (98), and GCN5 negatively impacts the functions of PGC1alpha, an important regulator of hepatic gluconeogenesis (99). Impairment of USP22 functions upon loss of SAGA integrity might also be related to the intrauterine retardation observed in the SUPT7L deficient lipodystrophy patient, as USP22 is required for placental vasculogenesis in mice (100). Hopefully future studies will provide additional insights as to how loss of SAGA functions are linked to functions of other factors associated with lipodystrophy and progeria.

## Connections to neural functions

In addition to the neural tube closure defects observed in mouse embryos bearing mutations in the GCn5 catalytic center, SAGA functions have been linked to neurodegeneration and to long-term memory consolidation.

PolyQ expansions in ATXN7, which docks the SAGA deubiquitinase module into the complex, give rise to spinocerebellar ataxia type 7 (SCA7). The impact of polyQ expansions in neural degeneration and the specific features of SCA7 are well-described in previous review articles (101). Molecularly, polyQ expansions in Atxn7 do not directly affect the integrity (102) or the DUB activity of SAGA, but they do trigger sequestration of SAGA into nuclear foci, impairing deubiquitination of chromatin associated targets, including H2Bub1 (103). GCN5 deficiency exacerbates the phenotype of a SCA7 mouse model, further indicating that reduction in SAGA functions contributes to the disease (104). How loss of USP22/DUB module function impacts degeneration of Purkinje cells or glia within the cerebellum is not yet clear, but ongoing work to delete USP22 specifically in these cells will hopefully provide insights.

Both histone acetylation and the production of acetyl-CoA have been linked to memory (105, 106, 107). Strikingly, among 18 KATs examined, GCN5/KAT2A was the most highly expressed in the hippocampal CA1 region, which is centrally important for learning and memory, in adult mouse brains (105). Loss of GCN5 specifically in excitatory neurons of the adult forebrain had no impact on overall brain morphology or on short-term memory. However, these mutant mice exhibited defects in long-term memory consolidation associated with impaired hippocampal synaptic plasticity. Molecularly, these changes in long-term memory were linked to changes in expression of genes involved in neuroactive receptor signaling upon loss of GCN5. Interestingly, acetylation has now been linked to different types of memory formation and cognition, further highlighting the potential for therapeutic interventions targeting these enzymes (108).

## Final thoughts

The field has come a long way since the discovery of Gcn5 as the first transcription-related KAT in 1996. Our understanding of the functions of GCN5, PCAF, and their associated complexes in gene regulation have expanded to include many steps in the process of transcription. We also now know that these enzymes have many nonhistone substrates, and that they impact many processes in addition to gene regulation. Both SAGA and ATAC have been linked to human disease, and future studies will no doubt uncover additional connections between these KATs and human maladies, as well as development of new therapies targeting these enzyme complexes. A single review cannot cover all of these discoveries, but the breadth of GCN5 and PCAF functions serve as an ongoing tribute to the many innovations of Dave Allis, whose discoveries and pure love of science enabled so many advances over the past 28 years.

## Conflict of interest

The author declares that they have no conflicts of interest with the contents of this article.
